# Rise and Decay of the COVID-19 Epidemics in the USA and the State of New York in the First Half of 2020: A Nonlinear Physics Perspective Yielding Novel Insights

**DOI:** 10.1155/2021/6645688

**Published:** 2021-05-18

**Authors:** Till D. Frank

**Affiliations:** ^1^Department of Psychological Sciences, University of Connecticut, Storrs, CT 06269, USA; ^2^Department of Physics, University of Connecticut, Storrs, CT 06269, USA

## Abstract

As of December 2020, since the beginning of the year 2020, the COVID-19 pandemic has claimed worldwide more than 1 million lives and has changed human life in unprecedented ways. Despite the fact that the pandemic is far from over, several countries managed at least temporarily to make their first-wave COVID-19 epidemics to subside to relatively low levels. Combining an epidemiological compartment model and a stability analysis as used in nonlinear physics and synergetics, it is shown how the first-wave epidemics in the state of New York and nationwide in the USA developed through three stages during the first half of the year 2020. These three stages are the outbreak stage, the linear stage, and the subsiding stage. Evidence is given that the COVID-19 outbreaks in these two regions were due to instabilities of the COVID-19 free states of the corresponding infection dynamical systems. It is shown that from stage 1 to stage 3, these instabilities were removed, presumably due to intervention measures, in the sense that the COVID-19 free states were stabilized in the months of May and June in both regions. In this context, stability parameters and key directions are identified that characterize the infection dynamics in the outbreak and subsiding stages. Importantly, it is shown that the directions in combination with the sign-switching of the stability parameters can explain the observed rise and decay of the epidemics in the state of New York and the USA. The nonlinear physics perspective provides a framework to obtain insights into the nature of the COVID-19 dynamics during outbreak and subsiding stages and allows to discuss possible impacts of intervention measures. For example, the directions can be used to determine how different populations (e.g., exposed versus symptomatic individuals) vary in size relative to each other during the course of an epidemic. Moreover, the timeline of the computationally obtained stages can be compared with the history of the implementation of intervention measures to discuss the effectivity of such measures.

## 1. Introduction

Within less than one year, the coronavirus disease 2019 (COVID-19) pandemic has claimed more than 1 million lives [1]. In view of this tragic number, it is important to study the regions and periods in which the COVID-19 epidemics at least regionally and temporarily subsided. In this context, studying the rise and decay of COVID-19 epidemics from the related perspective of nonlinear physics and synergetics allows to address the emergence and subsiding of the disease in a unifying way that helps to understand the possible impacts of COVID-19 intervention measures. According to the World Health Organization, the COVID-19 pandemic originated from Wuhan city, China, in December 2019 [2]. The disease spread quickly through China and beyond. As of the end of the year 2020, the disease has affected nations on all seven continents of the world [1]. Intervention measures ranging from face mask mandates to the lockdown of businesses have been put in place worldwide and have shown the potential to slow down the spread of the disease [3–6]. In this context, epidemiological measures and parameters have been considered such as the reproduction number and the effective contact rate, where the reproduction number is defined as the expected number of infected individuals directly generated by a typically infected person [7], and the effective contact rate is the rate with which effective contacts (contact leading to an infection) are made. A decrease of the reproduction number or the effective contact rate due to the implementation of an intervention measure indicates that the measure at hand works successfully. In fact, various studies have used those measures in order to shown that the implementation of intervention measures indeed has reduced the spread of COVID-19 [4, 8, 9]. In particular, it has been suggested to consider so-called active intervention policies that permanently monitor the infection status of a population and adjust continuously the intervention measures according to the observed status [10, 11]. With the help of such a dynamic feedback system, it is possible to make sure that the health care system of a country under consideration is not overburdened by the hospitalization of COVID-19 patients. However, the COVID-19 pandemic is affected by many factors [12, 13] including environmental factors such as the level of air pollution [12]. In particular, the particulate matter levels in the air may have an effect on the infection dynamics [13]. When considering the implementation of intervention measures, in general, and the shutdown of businesses, in particular, one should keep in mind the typically negative effects on the economics [14].

**Figure 1:**
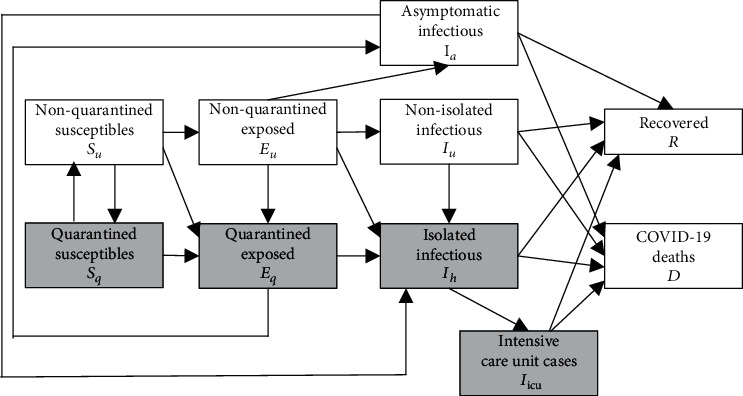
Infectious disease model developed in Ref. [24].

Among the many ways to describe the time course of COVID-19 cases (e.g., by means of autoregressive integrated moving average models [15]), epidemiological compartment models such as susceptible-exposed-infected-recovered (SEIR) models [16, 17] have been important tools to understand COVID-19 data and to examine the impact of intervention measures. Such SEIR models and generalized SEIR models have been used to examine COVID-19 epidemics in various countries around the globe [18–23]. For the current work, the study by Ngonghala et al. [24] is of particular interest. In this study, a generalized SEIR model was developed. The model involves nonquarantined and quarantined individuals. Accordingly, it accounts for susceptible (*S*_*u*_), exposed (*E*_*u*_), and symptomatic infectious (*I*_*u*_) nonquarantined or nonisolated individuals and susceptible (*S*_*q*_), exposed (*E*_*q*_), and symptomatic infectious (*I*_*h*_) quarantined or isolated individuals. For susceptible and exposed individuals, the attributes quarantined versus nonquarantined are used. In contrast, for symptomatic infectious individuals, the attributes isolated versus nonisolated are used. In particular, hospitalized symptomatic individuals belong to the compartment *I*_*h*_, whence the subindex “h.” Note that the compartment of isolated symptomatic individuals does not contain intensive care unit (ICU) cases. The model describes ICU COVID-19 patients (*I*_icu_) as their own class. Moreover, asymptomatic infectious COVID-19 cases (*I*_*a*_) constitute a compartment of their own. Finally, the model involves the compartment (*R*) of individuals recovered from COVID-19 and the compartment of COVID-19 associated deaths (*D*). The infectious compartments are *I*_*u*_, *I*_*a*_, and *I*_*h*_.  Figure 1 presents the compartments and a flow diagram describing the transitions of individuals between the compartments. For the sake of brevity, the phrase symptomatic infectious individuals will be abbreviated by symptomatic individuals.

As indicated in Figure 1, nonquarantine susceptibles (*S*_*u*_) get into contact with an individual of one of the infectious compartments and make one of three possible transitions. They get infected such that they become exposed individuals and are quarantined (*E*_*q*_). They get infected such that they become exposed individuals but are not quarantined (*E*_*u*_). They do not get infected but are quarantined (*S*_*q*_). The quarantined susceptible individuals (*S*_*q*_) either leave quarantine and become nonquarantined susceptibles (*S*_*u*_) or they are infected during quarantine and become quarantined exposed individuals (*E*_*q*_). Nonquarantine exposed individuals (*E*_*u*_) either become quarantined exposed individuals (*E*_*q*_), isolated symptomatic individuals (*I*_*h*_), nonisolated symptomatic individuals (*I*_*u*_), or asymptomatic infectious individuals (*I*_*a*_). In contrast, quarantine exposed individuals (*E*_*q*_) either become isolated symptomatic individuals (*I*_*h*_) or asymptomatic infectious individuals (*I*_*a*_). As far as nonisolated symptomatic individuals (*I*_*u*_) are concerned, they either become isolated cases (*I*_*h*_), recover (*R*), or decease due to COVID-19 (*D*). In contrast, isolated symptomatic individuals (*I*_*h*_) either become ICU cases (*I*_icu_), recover (*R*), or decease due to COVID-19 (*D*). ICU cases either recover (*R*) or decease due to COVID-19 (*D*). Finally, asymptomatic infectious individuals (*I*_*a*_) either recover (*R*), decease due to COVID-19 (*D*), or develop symptoms and become isolated symptomatic cases (*I*_*h*_). As mentioned above, the model by Ngonghala et al. [24] is aimed at distinguishing between nonquarantined or nonisolated individuals, on the one hand, and quarantined or isolated individuals, on the other. To this end, in Figure 1, the compartments that refer to quarantined or isolated individuals have been shaded in gray.

**Figure 2:**
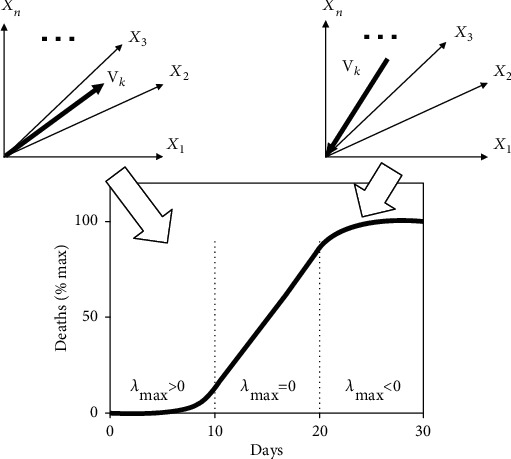
Three-stage model as developed in Ref. [32].

Epidemiological models as reviewed above have been analyzed from mathematical [25, 26] and numerical perspectives [27]. However, relatively little emphasis has been put on the nonlinear physics underlying the emergence as well as the subsiding of those epidemics. Accordingly, in general, an infectious disease emerges in a population due an instability: the infection dynamical system evolves away from an unstable disease-free fixed point [28]. An illustrative example of a mechanical system exhibiting an instability (or an unstable fixed point) is a ball on the top of hill. In general, instabilities are characterized by stability parameters, called eigenvalues. Any instability exhibits at least one positive stability parameter (or eigenvalue) [29–31]. Within the nonlinear physics perspective [29], in general, and the framework of synergetics [30, 31], in particular, this stability parameter determines the disease outbreak under consideration. Using the synergetics framework, recently, a three-stage model for COVID-19 epidemic has been developed that demonstrates that both the emergence and the subsiding of a COVID-19 epidemic are determined by the aforementioned positive stability parameter [32]. Similar stage models have been used in a study on the COVID-19 epidemic in Italy [9] and in a multicountry study addressing the COVID-19 epidemics in China, the USA, and a number of European countries [6]. The three-stage model when applied to describe COVID-19 associated deaths is illustrated schematically in Figure 2. The main panel in Figure 2 shows schematically the time course of the cumulative COVID-19 associated deaths (here, over a period of one month) during the three model stages of an epidemic under consideration. The stages are denoted by S1, S2, and S3 and are characterized by an exponential increase (S1), a linear increase (S2), and a deaccelerating increases (S3) of COVID-19 deaths. The underlying circumstances of the infection dynamical system that produce those three characteristic dynamical regimes can be described in terms of the largest (or maximal) stability parameter of the system (denoted by *λ*_max_). The first stage (S1) with exponentially increasing COVID-19 deaths is consistent with a positive maximal parameter, the linear stage (S2) is consistent with a maximal parameter equal to zero, and the subsiding stage (S3) with a maximal parameter that assumes a negative number. The two inserts on the top of Figure 2 illustrate schematically the characteristic dynamics in the outbreak (S1) and subsiding (S3) stages as seen in the epidemiological state space under consideration. In general, a *n*-dimensional state space spanned by *n* variables *X*_1_, ⋯, *X*_n_ is considered. Under appropriate conditions, in stage 1, there is a specific direction related to the maximal positive stability parameter *λ*_max_. This direction can be described with the help of a vector **v**_*k*_ that is called an eigenvector. In general, a system described in an *n*-dimensional state space can exhibit up to *n* different stability parameters that come with *n* different directions (i.e., eigenvectors), which constitute the key directions of the system at hand. As far as the specific vector **v**_*k*_ related to the positive stability parameter is concerned, the vector typically is referred to as unstable eigenvector because it is related to an instability [29–31]. As long as the initial epidemiological state is sufficiently close to the disease-free state, based on general theoretical considerations [31, 32], it follows that the infection dynamics will evolve during the COVID-19 outbreak along that particular direction. In a similar vein, under appropriate conditions, during the subsiding of the epidemic (S3), the direction **v**_*k*_ related to the maximal (now negative) stability parameter characterizes the infection dynamics. Accordingly, the infection dynamics subsides and converges to the disease-free state along the key direction specified by **v**_*k*_. In Ref. [32], the three-stage model was studied in the context of a two-dimensional epidemiological state space (i.e., the case *n* = 2 was considered). In the current study, a higher-dimensional state space will be considered.

Using the aforementioned three-stage modelling approach, the study [32] suggests that the lockdown intervention measures that were implemented during the spring months of 2020 in 15 out of 20 European countries induced a switch of the positive stability parameter of their epidemiological systems to a negative one. This switch will be called the sign-switching phenomenon. For those 15 countries, the COVID-19 free fixed point was stabilized, and the daily new infections decayed towards relatively low levels [32].

In the present study, the three-stage modelling approach is used to analyze the epidemics in the state of New York and nationwide in the USA during the first half of the year 2020, in which for both regions the infection dynamics exhibited a first-wave character. The 10-variable model by Ngonghala et al. [24] reviewed above will be used. The three-stage analysis demonstrated schematically in Figure 2 will be conducted in a five-dimensional subspace of the model (i.e., the case *n* = 5 will be considered). The model-based analysis will demonstrate that the first-wave epidemics in those regions showed the sign-switching phenomenon reported from the aforementioned European countries. It will be argued that this kind of stabilization was at least in part due to the intervention measures implemented in the state of New York and nationwide in the USA. In doing so, the study goals are twofold. The study is aimed at analyzing the rise and decay of the COVID-19 epidemics in the USA and the state of New York in the first half of the year 2020 by combining epidemiological modelling and nonlinear physics. In addition, within this combined approach, the study is aimed at addressing the efficacy of intervention measures.

## 2. Material and Methods

### 2.1. Data

The study evaluated data of COVID-19 associated deaths occurring regional in the state of New York and nationwide in the USA. COVID-19 death data are from Ref. [33].

### 2.2. Measures

Following Ref. [24], the current study considered two related measures: cumulative COVID-19 associated deaths and daily deaths.

### 2.3. Model Formulation

In the current study, the generalized SEIR model developed in Ref. [24] and described in the introduction (see also Figure 1) was used to describe the time course of the COVID-19 epidemics in the state of New York and nationwide in the USA. For the present study, the recovered individuals can be neglected as will be clear below. The evolution equations read [24] as follows:

(1) $$\begin{array}{c} \frac{dS_{u}}{dt}=-F\;S_{u}+k_{1}\;S_{q}, \end{array}$$

(2) $$\begin{array}{c} \frac{d\;S_{q}}{dt}=\left(1-p\right)FS_{u}-\left(\theta_{j}F+k_{1}\right)S_{q}, \end{array}$$

(3) $$\begin{array}{c} \frac{d\;E_{u}}{dt}=\left(1-q\right)pFS_{u}-k_{2}\;E_{u}, \end{array}$$

(4) $$\begin{array}{c} \frac{dE_{q}}{dt}=qpFS_{u}+\alpha E_{u}+\theta_{j}F\;S_{q}-k_{3}\;E_{q}, \end{array}$$

(5) $$\begin{array}{c} \frac{dI_{u}\;}{dt}=f_{1}\sigma_{u}\;E_{u}-k_{4}\;I_{u}, \end{array}$$

(6) $$\begin{array}{c} \frac{dI_{h}\;}{dt}=f_{2}\sigma_{u}\;E_{u}+r\sigma_{q}\;E_{q}+\phi \;I_{u}+\sigma_{a}I_{a}-k_{5}\;I_{h}, \end{array}$$

(7) $$\begin{array}{c} \frac{dI_{a}\;}{dt}=\left(1-f_{1}-f_{2}\right)\sigma_{u}\;E_{u}+\left(1-r\right)\sigma_{q}E_{q}-k_{6}\;I_{a}, \end{array}$$

(8) $$\begin{array}{c} \frac{d\;I_{icu}}{dt}=\upsilon \;I_{h}-k_{7}\;I_{icu}, \end{array}$$

(9) $$\begin{array}{c} \frac{dD}{dt}=\delta_{u}\;I_{u}+\delta_{h}\;I_{h}+\delta_{a}\;I_{a}+\delta_{\text{icu}}\;I_{\text{icu}}. \end{array}$$

The model equations (1) to (4) involve the force of infection *F* [16] defined by [24].

(10) $$\begin{array}{c} F=\beta \frac{\left(I_{u}+\eta_{a}\;I_{a}+\eta_{h}\;I_{h}\right)}{N-\theta_{q}\left(E_{q}+I_{h}+I_{\text{icu}}\;\right)}. \end{array}$$

In equation (10), the variable *N* is the population size. In the present study, following the arguments of previous works [21, 32, 34], variations in *N* over time were neglected since the observation period was relatively short and the variable *D* at all times was much smaller than *N*. Equations (1) to (10) involve the parameters *p*, *q*, *f*_1_, *f*_2_, *θ*_*j*_, *θ*_*q*_, *α*, *σ*_*u*_, *σ*_*q*_, *ϕ*, *r*, *ν*, *δ*_*u*_, *δ*_*h*_, *δ*_*a*_, *δ*_icu_, *η*_*a*_, *η*_*h*_, *k*_1_,…,*k*_7_, and *β*, which are semipositive. Importantly, in Ref. [24], the parameter *β* was used as an effective contact rate measure that takes impacts of intervention measures like physical distancing into account. However, in Ref. [24], the impact of wearing a face mask was described by a separate mathematical factor. In contrast, in the present study, the parameter *β* includes impacts of all kind of intervention measures (including wearing face masks) that lower the probability of an infection. For sake of completeness, the description of all other parameters together with their values is given in Table 1. For New York state, *N* = 19,400,000 was used. For the USA, we put *N* = 331,000,000. A detailed description of the parameters can be found in Ref. [24].

The disease-free fixed point of the model defined by equations (1) to (10) is the state for which the solutions do not change over time and for which there are neither exposed nor infectious individuals in the population. In order to study the outbreak of a novel infectious disease such as COVID-19, it is typically assumed that the whole population is susceptible. Consequently, the disease-free fixed point is given by *S*_*u*_ = *N* and *S*_*q*_ = *E*_*u*_ = *E*_*q*_ = *I*_*u*_ = *I*_*h*_ = *I*_*a*_ = *I*_icu_ = *D* = 0. In general, fixed points can be asymptotically stable, neutrally stable, or unstable [29]. For the current study, only the neutrally stable and unstable cases are relevant. A fixed point is unstable when the infection dynamics abandons the fixed point [29–31] (recall the mechanical example of a ball placed on the top of hill). In contrast, in the case of a stable fixed point, the infection dynamics converges towards the fixed point. A mechanical illustration of such a fixed point is a marble at the bottom of a bowl. For a neutrally stable fixed point, there is at least one dimension that features a continuous set of possible states to which the infection dynamics can converge. A mechanical example of a neutrally stable fixed point is a marble sitting at some point at the bottom of a horizontally oriented pipe. Since in what follows a relatively short period will be considered, this dimension is given by the nonquarantined susceptible individuals *S*_*u*_. When the epidemic has completely subsided such that all infectious and exposed individuals have either recovered or have deceased from COVID-19, there is not a particular value that *S*_*u*_ can assume but *S*_*u*_ can assume any value in an appropriately defined interval (just as the aforementioned marble in the pipe can rest at any point along the bottom of the pipe). In the rest of the paper, stable will be understood as neutrally stable for the sake of brevity. From previous work [24], it follows that the disease-free fixed point is unstable if the effective contact rate *β* is larger than a critical value *β*crit and stable if *β* is smaller than the critical value (for similar considerations see also Ref. [21, 32]). The critical value can be computed from

(11) $$\begin{array}{c} \beta \text{crit}=\frac{k_{2}k_{4}k_{5}k_{6}}{p\left(B_{u}+\eta_{a}\;B_{a}+\eta_{h}\;B_{h}\right)}, \end{array}$$

where *B*_*u*_, *B*_*a*_, and *B*_*h*_ are expressions of the model parameters that can be found in Ref. [24]. As such, the epidemiological model defined by (1) to (10) is a nonlinear dynamical system. In order to study the dynamics close to the disease-free fixed point, the nonlinearities can approximately be modelled by means of linear functions [30, 31]. The linearized dynamical system thus obtained can be described in terms of a linearization matrix. This matrix characterizes the dynamical system with respect to the original axes of the state space under consideration (e.g., the axes labelled *X*_1_, ⋯, *X*_*n*_ in the inserts of Figure 2). However, a new set of axes can be defined in terms of the aforementioned key directions or eigenvectors (such as the vector **v**_*k*_ shown in Figure 2). In doing so, the matrix characterizing the dynamics with respect to those new axes assumes the simple form of a diagonal matrix. The diagonal elements define mathematically the aforementioned stability parameters or eigenvalues [30, 31]. When carrying out this approach, by linearizing the model equations (1) to (10) at the disease-free fixed point, the relevant 9 by 9 linearization matrix can be obtained. For the stability of the fixed point, the dynamics of the variables *I*_icu_ and *D* do not play a role because they do not feedback into the linearized versions of equations (1) to (7). Therefore, the relevant linearization matrix is a 7 by 7 submatrix for the variables *S*_*q*_, *S*_*u*_, *E*_*u*_, *E*_*q*_, *I*_*u*_, *I*_*h*_, and *I*_*a*_. From the matrix, the stability parameters characterizing the dynamics close to the fixed point and the fixed point as such can be determined. The 7 by 7 matrix exhibits one parameter equal to zero due to the fact that demographic terms in the model are neglected [21]. This results in the aforementioned neural stability with respect to the variable *S*_*u*_. The 7 by 7 submatrix also exhibits the stability parameter −*k*_1_, which reflects that in the absence of any exposed or infectious individuals the dynamics of *S*_*q*_ is given by *dS*_*q*_/*dt* = −*k*_1_ *S*_*q*_, see equation (2). That is, the quarantined susceptibles decay exponentially because they leave quarantine. Consequently, the stability of the fixed point is eventually determined by the remaining 5 stability parameters. They can be obtained from a 5 by 5 submatrix related to the dynamics of the variables *E*_*u*_, *E*_*q*_, *I*_*u*_, *I*_*h*_, and *I*_*a*_. The 5 by 5 matrix reads

(12) $$\begin{array}{c} L=\left(\begin{array}{ccccc} -k_{2} & 0 & \left(1-q\right)p\beta & \left(1-q\right)p\beta \eta_{h} & \left(1-q\right)p\eta_{a}\\ \alpha & -k_{3} & qp\beta & qp\beta \eta_{h} & qp\beta \eta_{a}\\ f_{1}\;\sigma_{u} & 0 & -k_{4} & 0 & 0\\ f_{2}\;\sigma_{u} & r\sigma_{q} & \phi & -k_{5} & \sigma_{a}\\ \left(1-f_{1}-f_{2}\right)\sigma_{u} & \left(1-r\right)\sigma_{q} & 0 & 0 & -k_{6} \end{array}\right). \end{array}$$

Note that stability parameters may assume complex numbers that are composed of real and imaginary parts [29]. With the help of the stability parameters, stability can be mathematically defined. If the matrix *L* exhibits a positive stability parameter or a parameter featuring a positive real part, then the disease-free fixed point is unstable. If all parameters are negative or have negative real parts, then the disease-free fixed point is stable. From the aforementioned considerations, it follows that for *β* larger than *β*crit the 5 by 5 matrix exhibits at least one positive parameter (or a parameter with positive real part), whereas for *β* smaller than *β*crit all stability parameters are negative (or exhibit negative real parts). In the special case when *β* equals *β*crit, there is at least one parameter that equals zero, which is the largest parameter. Consequently, the three cases (i) *β* > *β*crit, (ii) *β* = *β*crit, and (iii) *β* < *β*crit correspond to the three cases (i) *λ*_max_ > 0, (ii) *λ*_max_ = 0, and (iii) *λ*_max_ < 0 of the three-stage model reviewed in the introduction, see also Figure 2. In other words, they correspond to the three stages S1, S2, and S3. In this context, note that the critical condition (ii), for which *λ*_max_ = 0 holds, is referred to as the bifurcation point of the system. Importantly, from dynamical systems theory [29] and the theory of synergetics [30, 31], it follows that slightly above the bifurcation point (i.e., *β* is slightly larger than *β*crit), there exist a key direction **v**_*k*_ (see Figure 2 again) related to the positive stability parameter *λ*_max_ that dominates the infection dynamics [21, 32] as discussed in the introduction. This direction is referred to as the order parameter of the COVID-19 outbreak [21, 30, 31]. The direction **v**_*k*_ can be tracked through different stages of an epidemic (see Results). In particular, when the epidemic subsides (presumably due to the impact of interventions), the vector **v**_*k*_ can describe the dominant direction of the decaying infection dynamics in the epidemiological 5 variable spaces (see Results).

### 2.4. Model-Based Data Analysis

Following the two studies reviewed in the introduction, namely, (i) the study [32] on a three-stage scheme of the first-wave COVID-19 epidemics in 20 European countries and (ii) the study [24] in which COVID-19 associated deaths have been used for fitting the models (1)-(10), the three-stage scheme was used to fit COVID-19 associated deaths from the state of New York and the USA to the model defined by equations (1) to (10). As mentioned above, data were obtained from Ref. [33]. As illustrated in Figure 2, the three-stage scheme [32] consists of an outbreak stage (stage 1), a critical stage (stage 2), and a final stage (stage 3). From the previous discussion, it follows that in the outbreak stage, *β* is assumed to be larger than *β*crit, the disease-free fixed point is unstable, and the cases and deaths increase more or less exponentially. In the critical stage, the COVID-19 epidemic is brought to some extent under control such that *β* is close to the critical value *β*crit and the fixed point is about to become stable. Note that in this stage the course of an epidemic typically exhibits a linear increase rather than an exponential one [32]. In the final stage, *β* drops below *β*crit, the COVID-19 free fixed point is stabilized (presumably due to intervention measures), and the epidemic subsides. For more details, see Ref. [32]. As in Ref. [24], COVID-19 associated deaths were considered starting March 1, 2020. Unlike Ref. [24], the whole 4 months period from March 1 to June 30 describing the first half of the year 2020 was considered. During that period, both the data from the state of New York and the nationwide USA data showed first-wave epidemics (see Results). As in Ref. [32], the parameter *β* was estimated for stages 1 and 3 using a standard nonlinear fitting procedure to minimize the error between the model predicted deaths (as described by the variable *D*) and the observed deaths, while for stage 2 the parameter *β* was fixed at the critical value *β*crit. All other model parameters were taken from Ref. [24] and are listed in Table 1. Note that the time points *t*_1_ and *t*_2_ of the beginning of stages 2 and 3 were varied to find the optimal time points that produced the best fit between model predicted and observed deaths. Since the time points *t*_1_ and *t*_2_ were derived from data, the analysis is regarded as a data-driven approach. Stability parameters (i.e., eigenvalues) were determined from the matrix *L* defined by equation (12). The COVID-19 outbreak key directions (i.e., order parameters) were determined from matrix *L* in stage 1. Likewise, the key directions (i.e., eigenvectors) determining the COVID-19 subsiding were determined from matrix *L* in stage 3.

## 3. Results and Discussion

**Figure 3:**
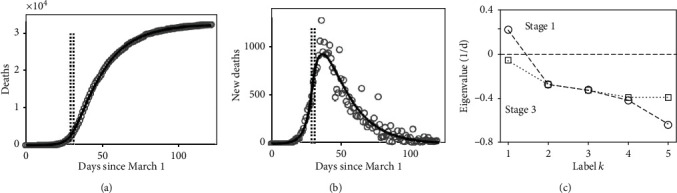
State of New York data and model-based analysis: (a) cumulative deaths as reported (gray circles) and predicted by the epidemiological three-stage model (solid black line); (b) new daily deaths (reported and fitted); (c) eigenvalues (i.e., stability parameters) of the matrix *L* defined by equation (12) in stages 1 (circles) and 3 (squares).

Figure 3 shows the data and modelling results for the state of New York. Panel (a) presents the cumulative confirmed deaths (gray circles) in the 4 months period from March 1 to June 30. The model fit is shown as well (solid black line). The model captured the characteristic sigmoid shape of the trajectory. The two vertical lines indicate the time points *t*_1_ and *t*_2_ of the beginning of stages 2 and 3 with *t*_1_ = 29 days (March 30) and *t*_2_ = 31 days (April 1). Panel (b) shows the daily new deaths as reported (gray circles) and obtained from the model (solid black line). The time points *t*_1_ = 29 days and *t*_2_ = 31 days are indicated as well, again, by vertical lines. Taking panels (a) and (b) together, the model-based analysis suggests that stage 2 was relatively short. Accordingly, COVID-19 emerged in March 2020 in the state of New York due to an instability (see below), and the outbreak was characterized by a dramatic increase in COVID-19 associated deaths. However, the infection dynamics changed within March such that at the end of March the unstable disease-free fixed point was about to become stable. The critical stage 2 was relatively short. Beginning of April, the disease dynamics entered stage 3. The disease-free fixed point was stabilized. Taking a conservative point of view, the analysis suggests that at least in May and June the fixed point was stable. Panel (b) demonstrates the delay of the stabilization effect on the trajectory of daily deaths. The tragic peak of about 1000 new deaths per day occurred around days 35 to 40 (April 5 to April 10), that is, a few days after the infection dynamics entered stage 3.

Panel (c) of Figure 3 shows the stability parameters (i.e., eigenvalues) obtained from the matrix *L* defined by equation (12) for stages 1 (outbreak stage) and 3 (subsiding stage). As expected, stage 1 was characterized by a set of values (circles connected by solid lines) that showed a positive stability parameter (here labelled *k* = 1), whereas stage 3 showed a set of parameters (squares connected by a dotted line) that were either real-valued and negative or had negative real parts. This illustrates that (as expected) the disease-free fixed point was unstable in stage 1 and stable in stage 3. Note that in stage 1, all stability parameters were real-valued. In contrast, stage 3 showed a pair of complex-valued parameters (*k* = 4 and *k* = 5). The real parts of such parameters are equal [29–31] as can be seen in panel (c). Note that panel (c) only presents the real parts of the stability parameters. Imaginary parts are not shown. Qualitatively, panel (c) shows the important sign-switching phenomenon that is required for an epidemic to end [32]: a positive stability parameter describing the instability of a virus-free epidemiological state of a population under consideration turns into a negative one. In this context, the estimated effective contact rates for stages 1 and 3 were *β* = 1.81/d and *β* = 0.23/d, respectively, with a critical value of *β*crit = 0.43/d. As expected, *β* was larger than the critical value in stage 1 and smaller than the critical value in stage 3.

In the first week of March 2020, the state of New York confirmed the first COVID-19 cases. In the same week, the first COVID-19 associated deaths were reported [33]. COVID-19 cases increased rapidly and so did the number of COVID-19 associated deaths. In particular, around March 20, the death toll passed the 100 mark. As a reaction to the COVID-19 outbreak, intervention measures were implemented step-by-step. A few milestones are the declaration of the state of emergency for the state of New York on March 7 [35], the prohibition of gatherings involving more than 500 people on March 12 [36], the partial, regional lockdown of New York City on March 16 [37] that involved public school closures and the closure of bars and restaurants, and finally the full state-wide lockdown on March 20 [38] that involved a state-wide stay-at-home order and effectively the closure of all businesses except for essential businesses such as food shops and pharmacies. This timeline of events matches to some extent with the model-based data-driven analysis of the COVID-19 epidemic in the state of New York reported above. As reported above, around the end of March and the beginning of April, the disease dynamics switched from stage 1 to stages 2 and 3. The partial lockdown of New York City on March 16 and the state-wide lockdown on March 20 took place two weeks and one week earlier, respectively. In line with the literature on the effectiveness of intervention measures [3–5, 39], it is plausible to assume that at least in part the intervention measures contributed to the qualitative change of the infection dynamics from a dynamics characterized by an unstable COVID-19 free fixed point to a dynamics characterized by a stable COVID-19 free fixed point. In other words, it is plausible to assume that the decay of the effective contact rate *β* below the critical value *β*crit and related to that the sign-switching of the parameter *k* = 1 (i.e., *λ*_max_) from a positive to a negative sign was at least in part caused by the intervention measures implemented in New York City and the state of New York.

**Figure 4:**
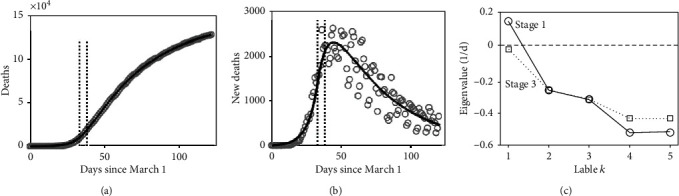
COVID-19 associated deaths reported from the USA from March 1 to June 30, 2020, and model-based analysis. (a–c) as in Figure 3.

Figure 4 summarizes the COVID-19 associated deaths reported from the entire USA and its model-based analysis. Just as in Figure 3, panel (a) of Figure 4 presents the cumulative confirmed deaths (gray circles) versus the model fit (solid black line). Again, the model adequately described the sigmoid shape of the trajectory. The time points *t*_1_ and *t*_2_ (indicated by vertical lines) were given by *t*_1_ = 33 days (April 3) and *t*_2_ = 38 days (April 8). Panel (b) shows the reported daily new deaths (gray circles) and the model fit (solid black line) with the time points *t*_1_ and *t*_2_ indicated as well. According to panels (a) and (b), on the nationwide scale of the USA, the stage 1 infection dynamics took place during the whole month of March and was characterized by a nationwide dramatic increase of COVID-19 associated deaths. Around the first week of April, the infection dynamics changed its character and entered stage 2, in which the fixed point was about to become stable. The model-based analysis suggests that stage 2 was relatively short (i.e., less than 1 week). The infection dynamics eventually entered stage 3, in which the fixed point was stabilized (at least from this large-scaled perspective that addresses the entire USA). According to our model-based analysis, the disease-free fixed point was stable at the end of the first half of the year 2020, that is, in May and June 2020. This is consistent with the more or less monotonic decay of the daily new deaths in May and June 2020 as shown in panel (b). Just as in the case of the analysis of the data from the state of New York, panel (b) of Figure 4 illustrates a delay of the effect of the stabilization of the COVID-19 free state on the daily new COVID-19 associated deaths. The daily death trajectory reached a high plateau of about 2500 deaths per day during the period from days 37 to 55 (April 7 to 30), although (at least according to our model-based analysis) the COVID-19 free fixed point was stabilized already around the beginning of that plateau (i.e., around April 8). Finally, note that second and third waves have been observed meanwhile in countries around the world. Such second and third waves have also been observed in the second half of the year 2020 on the nationwide scale of the USA, which, however, is beyond the scope of this study.

Panel (c) of Figure 4 shows the stability parameters for stages 1 and 3. Stage 1 featured one real-valued positive parameter *λ*_max_ (here labelled *k* = 1) indicating that the disease-free fixed point was unstable. In contrast, stage 3 featured a set of values that were all negative or exhibited negative real parts. Importantly, the parameter *k* = 1 (i.e., *λ*_max_) switched its sign from a positive to a negative value (sign-switching phenomenon [32]), demonstrating the change in stability of the disease-free fixed point from an unstable to a stable one. Note that both stages featured pairs of complex-valued parameters (labelled *k* = 4 and *k* = 5). The model-based analysis produced estimated effective contact rates of *β* = 1.38/d and *β* = 0.39/d for stages 1 and 3, respectively, with a critical value of *β*crit = 0.49/d. Consequently, *β* was larger than *β*crit in stage 1 and smaller than *β*crit in stage 3 (as expected), which is also consistent with the observed sign-switching phenomenon of *λ*_max_.

Early intervention measures on the large scale of the USA aimed to prevent SARS-CoV-2 infected individuals from entering the USA. To this end, travel restrictions against several countries such as China and the European countries were put in place [40, 41]. Nevertheless, as reported by the World Health Organization [42], on March 12, the epidemic in the USA had reached about 1000 confirmed cases and had claimed 29 lives. On March 13, President Donald Trump declared the national emergency to fight the COVID-19 epidemic in the USA [39]. The number of COVID-19 associated deaths continued to increase rapidly as can be seen in panel (a) of Figure 4. In April 2020, the USA became the country with the highest death toll worldwide [43]. By the end of March, most states of the USA had issued stay-at-home orders [44]. That is, the country was nationwide under lockdown. The timeline of those nationwide stay-at-home orders fits with the model-based data-driven analysis reported above that suggests that in the first week of April the nationwide epidemic was brought under control in the sense that (i) the epidemic switched from state 1 to stages 2 and 3, (ii) the effective contact rate dropped below the critical threshold, and (iii) the COVID-19 free epidemiological state was stabilized. That is, the intervention measures implemented in March 2020 probably contributed to those changes. In other words, by comparing the history of events about the implementation of intervention measures to the model-based data-driven identified epidemiological stages, it is plausible to assume that the implementation of intervention measures regionally in the state of New York and nationwide in the USA was the likely cause for the subsiding of the COVID-19 cases at the end of the first half of the year 2020.

In closing these considerations, let us note that while the deterministic model defined by Eqs. (1) to (10) can capture the temporal patterns of the observed trajectories shown in panels (a) and (b) of Figures 3 and 4, it does not account for stochastic aspects [45]. Stochastic aspects of the data become clearly visible when examining the daily new deaths as shown in panels (b) of Figures 3 and 4. In this context, for example, a SEIR Markov chain model has been suggested to study COVID-19 trajectories and the impacts of intervention measures within a stochastic framework [5].

Table 2 presents the key directions (i.e., eigenvectors) related to the largest stability parameters *λ*_max_ in stages 1 and 3 in the five-dimensional epidemiological space spanned by the variables *E*_*u*_, *E*_*q*_, *I*_*u*_, *I*_*h*_, and *I*_*a*_. Note that vectors are normalized to unity [30, 31]. Consequently, the magnitude of the vectors is not an issue. At issue is the direction in which they point in their respective state spaces. Table 2 shows the stage 1 vectors for the state of New York and the USA nationwide that are related to the positive stability parameter *λ*_max_ labelled *k* = 1 in panels (c) of Figures 3 and 4. In stage 1, the infection dynamics in the state of New York and the USA increased exponentially along the directions specified by the respective vectors [21, 30, 31]. In contrast, in all other directions of the five-dimensional (*E*_*u*_, *E*_*q*_, *I*_*u*_, *I*_*h*_, *I*_*a*_) space perturbations away from the disease-free fixed point decayed (because the stability parameters *k* = 2, ⋯, 5 were negative or exhibit negative real parts). Therefore, the stage 1 vectors shown in Table 2 determined the dynamics of the COVID-19 outbreaks in the state of New York and nationwide in the USA. They correspond to the vector **v**_*k*_ shown schematically in Figure 2. Using the terminology of synergetics [21, 30, 31, 46], they describe the order parameters of the COVID-19 epidemics in those two regions. Table 2 also presents the key directions (i.e., eigenvectors) of stage 3 related to the largest (but negative) parameter *λ*_max_ of stage 3 shown in panels (c) of Figures 3 and 4 at *k* = 1. These vectors describe directions along which the COVID-19 dynamics decayed towards the origin (see Figure 2 again), that is, the COVID-19 free state, in May and June. Importantly, the dynamics along those directions had the largest time constant relative to all other possible directions. That is, the dynamics in all other directions decayed relatively quickly towards the origin (because the stability parameters *k* = 2, ⋯, 5 or real parts were larger in the amount than the stability parameter with index *k* = 1). Therefore, the key dynamics that determined how fast the COVID-19 epidemics subsided was the dynamics along those directions related to the *k* = 1 stability parameter. Since those directions capture the stabilization stage, they have been referred to as stabilization directions in Table 2. As can be seen in Table 2, the COVID-19 order parameters both of the state of New York and the USA pointed strongly in the direction of the axis of the nonquarantined exposed individuals (*E*_*u*_). It points in the direction of the unknown or undetected exposed individuals. In contrast, the order parameters pointed only to a small degree in the direction of the nonisolated symptomatic infectious (*I*_*u*_) or asymptomatic infectious (*I*_*a*_) individuals. Accordingly, in March 2020, the epidemics both regionally in the state of New York and nationwide in the USA produced a more dramatic increase of exposed individuals as compared to nonquarantined symptomatic or asymptomatic individuals. The stabilization vectors that dominated (according to our model-based analysis) the infection dynamics in the state of New York and the USA in May and June 2020 pointed primarily in the direction of the isolated symptomatic infectious (*I*_*h*_) individuals. That is, the model-based analysis suggests that during May and June 2020, the decay of individuals of this compartment was relatively large as compared to the decay of individuals of the remaining four compartments. For illustration purposes of the meaning of the order parameters and stabilization directions, let us assume that during the outbreak and the subsiding stages, the infection dynamics was completely dominated by the order parameters and stabilization directions listed in Table 2. In this case, the weights (or coordinates) of components *E*_*u*_, *E*_*q*_, *I*_*u*_, *I*_*h*_, and *I*_*a*_ can be equated with individuals. Accordingly, during the outbreak in March 2020 in the state of New York and the USA, when the number of nonisolated symptomatic individuals (*I*_*u*_) increased by 12, then in the same period of time, the number of unobserved (i.e., nonquarantined) exposed individuals (*E*_*u*_) increased by 79 and 74, respectively. Roughly speaking, for every nonisolated symptomatic individual, seven nonquarantined exposed individuals showed up. Similarly, during the months of May and June characterized by subsiding epidemics in the state of New York and the USA, when the number of isolated symptomatic (e.g., hospitalized) individuals (*I*_*h*_) decayed by 80 and 75, respectively, then in the same period, the number of nonisolated symptomatic individuals (*I*_*u*_) decreased only by 10 and 12. Roughly speaking, for every 8 hospitalized (or otherwise isolated) patients who were cured (or deceased), the group of nonisolated symptomatic individuals only decayed by 1 person. COVID-19 order parameters have been previously been identified for the outbreaks in Wuhan city, China, and 22 administrative regions of Italy [21]. In this context, a standard four-variable SEIR model was used. For the data from Wuhan city, the vector describing stabilization direction was determined as well. Interesting, for the epidemic in Wuhan city, this vector characterizing the subsiding of the epidemic exhibited a larger coefficient for the exposed individuals as compared to the order parameter characterizing the epidemic outbreak. This relationship is just opposite to the relationship between the stabilization direction and the order parameter for the epidemics in the state of New York and the USA (see Table 2). Having said that, it is difficult to compare the two model-based analyses because the four-variable SEIR model used in Ref. [21] cannot be easily mapped to the generalized SEIR model described by equations (1) to (10).

The previous discussions reveal that the nonlinear physics perspective can be used to obtain a number of practical insights that can be summarized as follows:

- *Stage 2 as the Tipping Point Indicating That a Minimal Goal of Intervention Policies Has Been Achieved*. As pointed out by Wu et al. [47], intervention measures should minimally reduce the reproduction number (see Introduction) to a value of 1. This condition of an epidemic with reproduction number equal to 1 corresponds to the stage 2 of the three-stage model. Reaching this tipping point by means of the implementation of intervention measures indicates that the measures were successful in stopping the typically exponential increase of infections observed in the initial stage of an epidemic. The data-driven three stage approach rooted in the concepts of nonlinear physics allows to identify this tipping point. Note that while tipping points frequently correspond to undesired events, this is not the case in the current context. The tipping point of stage 2 has to be reached first [6, 32, 47]. Subsequently, the intervention measures should suppress the infection process under consideration even harder to initiate the subsiding of the infection dynamics
- *Speed of Changes in Group Sizes or Compartment Sizes*. The nonlinear physics approach yields a relatively simple way to determine the speed with which group sizes change relative to each other. Here, the groups are defined by the individuals of the compartments defined by epidemiological models (for examples, see above)
- *Set of Stability Parameters (Eigenvalues) Can Provide Supplementary Insights about the COVID-19 Dynamics*. Stability parameters may exhibit nonvanishing imaginary parts, which indicates that the infection dynamics is characterized by an oscillatory component. In fact, for the state of New York, such nonvanishing imaginary parts were identified for the COVID-19 dynamics of the state of New York during the subsiding stage (Figure 3(c)) and for the COVID-19 dynamics nationwide in the USA both during the outbreak and subsiding stages (Figure 4(c)). While in those examples, the oscillatory components did not make essential contributions to the trajectories under consideration (Figures 3(a) and 4(a)); in general, oscillatory components may make an essential contribution to the infection dynamics. If so, they may be difficult to distinguish from new outbreaks. Carrying out the analysis as shown above, may help to clarify, whether a sudden increase in a COVID-19 trajectory describes a new outbreak or is part of a rising or subsiding wave that exhibits an oscillatory component
- *Data-Driven Partitioning of COVID-19 Waves*. The approach separates COVID-19 waves into three stages guided by the general principles of dynamical systems theory and nonlinear physics that are assumed to determine the rise and decay of COVID-19 waves. Since the partitioning is data-driven, the approach reduces a possible interpretation-bias of researchers who attempt to interpret COVID-19 trajectories (see also Refs. [6, 32])

## 4. Conclusions

A key finding of this study is that the first-wave COVID-19 epidemics during the first half of the year 2020 observed nationwide in the USA and regionally in the state of New York emerged via an instability of the virus-human system and subsided via a stabilization of that instability. From the analysis presented above, the conclusion can be drawn that the three-stage model featuring an outbreak stage, a critical (linear) stage, and a subsiding stage can capture the key characteristics of first-wave epidemics not only for European countries (as demonstrated in an earlier study [32]) but also the USA and US states such as the state of New York. Having said that future studies have to work out the details for other US states beyond the state of New York. As far as research on the COVID-19 pandemic is concerned, the study results suggest that researchers exploit the tools of nonlinear physics in order to obtain insights into the nature of the infection dynamics during the outbreak and subsiding stages of COVID-19 waves. In particular, the study encourages future investigations to compare the history of events about the implementation of intervention measures with the timeline of data-driven identified epidemiological stages in order to address the efficiency of intervention measures.

## Figures and Tables

**Table 1:** Description of model parameters and values as reported in Ref. [24].

Parameter	Description	NY value	USA value
*p*	Probability of infection per contact	0.8073	0.7163
*q*	Proportion of being quarantined	0.2	Same as NY
*f*_1_	Proportion of exposed *E*_*u*_ who transition to *I*_*u*_	0.4	Same as NY
*f*_2_	Proportion of exposed *E*_*u*_ who transition to *I*_*h*_	0.2	Same as NY
*θ*_*j*_	Efficacy of quarantine	0.5	Same as NY
*θ*_*q*_	General efficacy of quarantine and isolation	1.0	Same as NY
*α*	Quarantine rate of exposed *E*_*u*_	0.1160/d	0.1065/d
*σ*_*u*_	1/*σ*_*u*_ is incubation period of exposed *E*_*u*_	0.1961/d	Same as NY
*σ*_*q*_	1/*σ*_*q*_ is incubation period of exposed *E*_*q*_	0.1961/d	Same as NY
*ϕ*	Isolation rate of *I*_*u*_	0.2/d	Same as NY
*r*	Proportion of exposed *E*_*q*_ who transition to *I*_*h*_	0.7	Same as NY
*ν*	Rate of progression to ICU case	0.083/d	Same as NY
*δ*_*u*_	Death rate of nonisolated infectious *I*_*u*_	0.015/d	Same as NY
*δ*_*h*_	Death rate of isolated infectious *I*_*h*_	0.015/d	Same as NY
*δ*_*a*_	Death rate of asymptomatic cases *I*_*a*_	0.0075/d	Same as NY
*δ*_icu_	Death rate of ICU patients	0.0225/d	Same as NY
*η*_*a*_	Reduced infectiousness for asymptomatic cases	0.5	Same as NY
*η*_*h*_	Reduced infectiousness for isolated cases *I*_*h*_	0.5	Same as NY
*k*_1_	Removal rate of *S*_*q*_	0.0714/d	Same as NY
*k*_2_	Removal rate of *E*_*u*_	0.3129/d	0.3026/d
*k*_3_	Removal rate of *E*_*q*_	0.1961/d	Same as NY
*k*_4_	Removal rate of *I*_*u*_	0.3150/d	Same as NY
*k*_5_	Removal rate of *I*_*h*_	0.2230/d	Same as NY
*k*_6_	Removal rate of *I*_*a*_	0.3908/d	0.4563/d
*k*_7_	Removal rate of *I*_icu_	0.1125/d	Same as NY

**Table 2:** Order parameters and stabilization directions characterizing the rise and decay of the COVID-19 epidemics during the first half of the year 2020 in the state of New York and nationwide in the USA.

	Stage 1		Stage 3	
	Order parameter		Stabilization direction	
Component	State of New York	USA	State of New York	USA
*E*_*u*_	0.79	0.74	0.34	0.45
*E*_*q*_	0.47	0.47	0.45	0.46
*I*_*u*_	0.12	0.12	0.10	0.12
*I*_*h*_	0.35	0.35	0.80	0.75
*I*_*a*_	0.15	0.15	0.16	0.14

## Data Availability

All data used in the study are publicly available from the website described in Ref. [33].
